# Complicated Iatrogenic Pneumopericardium in a Patient With Suspected Multiple Myeloma

**DOI:** 10.7759/cureus.24483

**Published:** 2022-04-25

**Authors:** Pedro A Adrover López, Darcy M Diago Blanco, Pedro Santiago Rodríguez, Brian González Sanabria, Rafael J Rivera Berrios

**Affiliations:** 1 Cardiovascular Disease Fellowship, Centro Médico Episcopal San Lucas, Ponce, PRI; 2 Internal Medicine, Centro Médico Episcopal San Lucas, Ponce, PRI; 3 Cardiovascular Disease, Centro Médico Episcopal San Lucas, Ponce, PRI

**Keywords:** iatrogenic complication, multiple myeloma, pneumopericardium, imaging, complication, echocardiography, tamponade, pericardial effusion

## Abstract

Pneumopericardium is the presence of air within the pericardial cavity. Though several etiologies and pathological mechanisms for this have been described, there is no clear consensus. Most resolve spontaneously, but others may develop severe complications such as cardiac tamponade which may lead to cardiorespiratory arrest. We report a rare case of a patient who developed a tension pneumopericardium requiring emergent aspiration following a therapeutic pericardiocentesis. Radiological and echocardiographic findings of this rare iatrogenic pneumopericardium are reviewed.

## Introduction

Pneumopericardium is a rare complication resulting from blunt or penetrating chest trauma, barotrauma, infections, air-containing fistulas between the pericardium and malignancies surrounding the mediastinum, as well as iatrogenic factors leading to accumulation of air in the pericardial cavity [1]. Patients can remain asymptomatic as long as the pneumopericardium does not cause compression on the heart, provoking diastolic dysfunction. Most of the cases are self-limiting and do not require specific treatment except for the close observation of hemodynamic stability, serial EKGs, and echocardiography. It is also important to treat concomitant pathologies. Timely diagnosis of this pathology is crucial in order to provide prompt lifesaving treatments.

Through this case, we intend to provide a thorough discussion of radiological and echocardiographic findings suggestive of pneumopericardium in order to bring awareness of iatrogenic pneumopericardium and its potentially fatal complications. Additionally, we highlight the importance of scrupulous procedural technique and the need for close monitoring following pericardiocentesis.

## Case presentation

We report a case of a 70-year-old man who visited his cardiologist due to progressive dyspnea on exertion for one week. The patient had a medical history of highly suspected multiple myeloma, gout, hypertension, atrial fibrillation, and non-ischemic cardiomyopathy. Physical examination revealed distant heart sounds, distended neck veins, rapid irregular pulse (105 beats/min), and blood pressure of 95/68 mmHg. For the past months, the patient had developed worsening renal function, vertebral lytic lesions, unexplained drops in hemoglobin levels, and elevated free kappa and lambda light chains. He was undergoing outpatient workup for suspected multiple myeloma. However, further investigations were delayed due to the development of a large pericardial effusion requiring emergent drainage of 700 mL of serous fluid about 1 month prior to the current visit. Former pericardial fluid analysis bared no evidence of malignancy.

**Figure 1:**
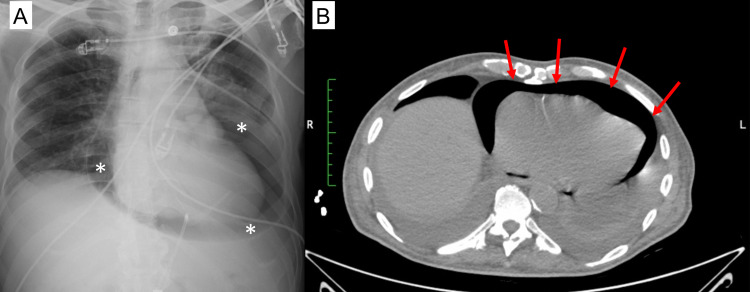
Post-Procedure Pneumopericardium Imaging (A) Portable chest X-ray with view of a large pneumopericardium (asterisks); (B) chest CT without contrast with cross-sectional view of pneumopericardium (arrows)

A bedside limited 2D echocardiography was performed and revealed a very large pericardial effusion provoking collapse of the right ventricular free wall during diastole (Supplemental Video 1). The patient was referred to the closest emergency department for emergent pericardiocentesis. A total of 1,300 mL of serous fluid was drained without any noticeable complication. Post-procedure surveillance with bedside echocardiography was impeded due to the inability to obtain a suitable sonographic window. A chest X-ray (Figure 1A) was requested and a subsequent chest computed tomography (CT) (Figure 1B) which exhibited a large amount of air within the pericardium. A tension pneumopericardium developed rapidly rendering the patient hypotensive and requiring invasive mechanical ventilation.

**Video 1:** Rare Iatrogenic Pneumopericardium: A 2D Echo Review—Diastolic Collapse Secondary to a Large Pericardial Effusion Transthoracic echocardiogram subxiphoid view showing diastolic collapse of the right ventricle secondary to a large pericardial effusion

After evacuating the pericardial air through the previously implanted pericardiocentesis drainage catheter, the patient became stable. Repeat echocardiography showed no evidence of diastolic compromise and follow-up chest X-rays demonstrated complete resolution of the pneumopericardium as well (Supplemental Video 2).

**Video 2:** Rare Iatrogenic Pneumopericardium: A 2D Echo Review—Resolution of Iatrogenic Pneumopericardium Transthoracic echocardiogram apical four-chamber view of heart following emergent pericardiocentesis with resolution of iatrogenic pneumopericardium

Despite hemodynamic improvement and no evidence of pneumopericardium recurrence, the patient remained in the intensive care unit due to worsening pleural effusions and respiratory failure on invasive mechanical ventilation. He was scheduled to continue outpatient follow-up with nephrology and oncology specialists if discharged but passed away one month after initial admission.

## Discussion

Pneumopericardium is a rare condition in which air enters the pericardial sac. Four major classifications for pneumopericardium development currently exist: dull or penetrating chest injury or barotrauma, fistulas between the pericardial sac and air-containing organs and structures, secondary production of gas by bacteria inhabiting the pericardium, and iatrogenic complications [1]. Even though multiple myeloma was suspected in our patient, the chronicity of events and imaging studies suggested that the tension pneumopericardium was iatrogenic in nature rather than a malignancy-related complication. However, multiple myeloma and its management strategies have been associated with recurrent pericardial effusions as well as bronchopericardial fistulas [2-4] which brings into question the extent to which malignancies can predispose patients to pneumopericardium formation.

Pericardiocentesis by catheter drainage under local anesthesia is the most common therapeutic intervention for symptomatic pericardial effusions. The subxiphoid approach is the standard technique to avoid complications. However, pericardiocentesis-related pneumopericardium is a rare complication that can occur in some cases; most likely due to a leaky drainage system or due to a pleuro-pericardial communication made during puncture [5]. In cases of hemodynamic worsening despite the evacuation of a pericardial effusion, iatrogenic pneumopericardium should be considered among differential diagnoses. The pneumopericardium seen in our case is likely due to a leaky drainage system, rather than a pleuro-pericardial communication, leading to the entrance of air into the pericardial sac. Patients with a puncture or fistulous tract-related pneumopericardium commonly develop life-threatening complications as well as evidence of recurrent pneumopericardium despite repeat manual evacuation of air from the pericardial sac [6,7]. If a leaky drainage system is suspected as a potential cause of pneumopericardium, immediate recognition and treatment of this rare complication are essential.

Clinical presentation can be symptomatic or asymptomatic, which can delay the diagnosis. The most common presenting symptoms of pneumopericardium are dyspnea and chest pain; however, other symptoms include fever, weight loss, hemoptysis, and cough. [8] In severe cases, patients can become hemodynamically compromised which may lead to cardiac arrest or, in some cases, death.

Conventional imaging modalities such as chest X-ray, CT, or echocardiography can confirm a pneumopericardium. Radiographic findings will describe the presence of a radiolucent halo covering the cardiac silhouette [9]. However, distinguishing pneumopericardium from pneumomediastinum, which is more common, can be challenging. If a chest X-ray includes air that envelops the aortic arch and superior vena cava above the azygous vein or distal left pulmonary artery, this is indicative of air outside the limits of the pericardium. [4] Some cases may require chest CT to evaluate pneumopericardium size and to rule out causes like fistulous communications between the esophagus or lungs as well as proof of other injuries that may lead to this rare complication [8,10,11]. In addition, echocardiographic findings that may suggest a pneumopericardium include an air gap sign where the image disappears when the heart contracts during systole (Supplemental Video 3), absence of an image (Supplemental Video 4), or a swirling bubble sign where multiple bright echoes pile up to form a rectilinear line which corresponds to the air-fluid level (Supplemental Video 5) [6,12].

**Video 3:** Rare Iatrogenic Pneumopericardium: A 2D Echo Review—Intermittent Loss of Signal with Systole Transthoracic echocardiogram parasternal lung axis view showing an inability to obtain a proper image due to intermittent loss of signal with systole secondary to a large iatrogenic pneumopericardium

**Video 4:** Rare Iatrogenic Pneumopericardium: A 2D Echo Review—Absence of an Image Transthoracic echocardiogram shows absence of an image secondary to a large iatrogenic pneumopericardium

**Video 5:** Rare Iatrogenic Pneumopericardium: A 2D Echo Review—“Bubble Sign” Transthoracic echocardiogram four-chamber view showing a classic “bubble sign” surrounding the left ventricle secondary to a large iatrogenic pneumopericardium

If air is identified within the pericardial sac without any signs of hemodynamic instability or cardiac tamponade, most symptoms of pneumopericardium will dissipate on their own. The strategy should aim toward treating the underlying cause. However, close monitoring of the patient’s clinical and radiological condition is required [1]. In other reported cases, authors oppose the performance of prophylactic decompression in all cases of pneumopericardium given two-thirds of patients do not develop cardiac tamponade [13].

Similar to our case, when there is evidence of a tension pneumopericardium, patient management should strive for pericardial decompression. The removal of excess air can be performed by pericardiocentesis or incision and drainage of the pericardial sac [7]. Once pericardial decompression is achieved, replacement of the leaky drainage system and prompt bedside echocardiography are essential to avoid repeat iatrogenic complications. Doing so will generally restore hemodynamic stability.

## Conclusions

Iatrogenic pneumopericardium can have a variety of presentations. Most asymptomatic cases are managed conservatively and usually resolve without any intervention. However, cases with features of cardiac tamponade require emergent drainage of air from the pericardial sac. These cases have a high mortality rate if not diagnosed and treated in a timely manner. The choice of treatment will depend on the etiologies leading to air accumulation within the pericardial sac and the stability of the patient’s condition. Diagnosis of a tension pneumopericardium can be ascertained with clinical and radiological findings. Our case is a prime example of how prompt identification of a large pneumopericardium can lead to lifesaving treatments.

## References

[1] Gołota JJ, Orłowski T, Iwanowicz K, Snarska J (2016). Air tamponade of the heart. Kardiochir Torakochirurgia Pol.

[2] Devasia AJ, Irodi A, George B (2016). Broncho-pericardial fistula leading to pneumopericardium following allogeneic stem cell transplantation. Indian J Pediatr.

[3] Jamison LS, Mo CC, Kwok M (2020). Pericardial relapse of multiple myeloma. BMJ Case Rep.

[4] Hirani S, Velez Martinez CS, Patan S, Kavanaugh M (2020). Cancer-related pneumopericardium: a case report and literature review. Case Rep Oncol.

[5] Tonial CT, Garcia PC, Victora J, Costa CA, Velasques JS, Martins MA (2019). Pneumopericardium: a rare case of cardiorespiratory arrest. Einstein (Sao Paulo).

[6] Schrank S, Khan MA, Flanagan M, Washco V, Kasarabada A (2021). Pyopneumopericardium: a case of a broncho-pericardial fistula from a cavitary lung mass. Chest.

[7] Lages J, Oliveira CC, Lacerda C (2018). Pneumopericardium due to bronchopericardial fistula in a patient with lung cancer. BMJ Case Rep.

[8] Chung TJ, Wang CK (2015). Pneumopericardium due to mediastinum metastatic lymph nodes. Heart Lung Circ.

[9] Sener YZ, Babacan T, Sarica MA, Turkbeyler IH (2013). A patient with pneumopericardium secondary to extranodal T-cell lymphoma. BMJ Case Rep.

[10] Liao HQ, Zhou D, Shang QL, Shi YF, Zhang M (2017). Pneumopericardium as a rare complication after esophagopericardial fistula. Echocardiography.

[11] Baird A, Gandhi M (2015). Pneumopericardium and pneumothorax due to right atrial permanent pacemaker lead perforation. J Med Imaging Radiat Oncol.

[12] Fadl SA, Nasrullah A, Harris A, Edwards R, Kicska G (2020). Comprehensive review of pericardial diseases using different imaging modalities. Int J Cardiovasc Imaging.

[13] Roth TC, Schmid RA (2002). Pneumopericardium after blunt chest trauma: a sign of severe injury?. J Thorac Cardiovasc Surg.

